# Epidemiological analysis of classical swine fever in wild boars in Japan

**DOI:** 10.1186/s12917-021-02891-0

**Published:** 2021-05-11

**Authors:** Yumiko Shimizu, Yoko Hayama, Yoshinori Murato, Kotaro Sawai, Emi Yamaguchi, Takehisa Yamamoto

**Affiliations:** Viral Disease and Epidemiology Research Division, National Agriculture and Food Research Organization, National Institute of Animal Health, Tsukuba, Ibaraki, Japan

**Keywords:** Classical swine fever (CSF), Japan, Wild boar, Oral bait vaccination

## Abstract

**Background:**

Classical swine fever (CSF) is a contagious disease of pigs and wild boars that is transmitted through direct/indirect contact between animals or CSF virus-contaminated fomites. When the disease re-emerged in 2018 in Japan, a CSF-infected wild boar was reported shortly after the initial pig farm outbreak; subsequently, the disease spread widely. To control the disease spread among wild boars, intensive capturing, fencing, and oral bait vaccination were implemented with concomitant virological and serological surveillance. This study aimed to describe the disease spread in the wild boar population in Japan from September 2018, when the first case was reported, to March 2020, based on the surveillance data. We conducted statistical analyses using a generalized linear mixed model to identify factors associated with CSF infection among wild boars. Moreover, we descriptively assessed the effect of oral bait vaccination, which started in March 2019 in some municipalities in the affected areas.

**Results:**

We observed a faster CSF infection spread in the wild boar population in Japan compared with the CSF epidemics in European countries. The infection probability was significantly higher in dead and adult animals. The influence of the multiple rounds of oral bait vaccination was not elucidated by the statistical modeling analyses. There was a decrease and increase in the proportion of infected and immune animals, respectively; however, the immunization in piglets remained insufficient after vaccination for 1 year.

**Conclusions:**

Conditions regarding the wild boar habitat, including forest continuity, higher wild boar population density, and a larger proportion of susceptible piglets, were addressed to increase the infection risk in the wild boar population. These findings could improve the national control strategy against the CSF epidemic among wild boars.

**Supplementary Information:**

The online version contains supplementary material available at 10.1186/s12917-021-02891-0.

## Background

Classical swine fever (CSF) is a contagious disease that affects pigs and wild boars. It is caused by a single-stranded RNA virus of the *Pestivirus* genus of the *Flaviviridae* family and is transmitted through direct/indirect contact between animals or virus-contaminated fomites. CSF is widely distributed in Asia, Africa, Central and South America, and Europe. Given its economic impact on pig production, its control has been among the priorities in pig-producing countries. In recent decades, CSF eradication has been achieved in the European Union, as well as some Central and South American countries [1–3]. Japan had achieved CSF eradication and was officially recognized as CSF-free in 2015 by the World Organisation for Animal Health [4]. However, it re-emerged in 2018, with reported infections in pig farms and wild boars [5, 6].

Generally, infectious disease control in wild animals requires considerable time and effort [7]. Control measures against infectious diseases aim to reduce the number of susceptible animals to mitigate disease spread within the affected animal population. The CSF epidemic in the wild boar population had been mostly reported in Europe since the 1980s, with eradication being achieved after more than 20 years [8, 9]. During this European epidemic, measures such as fencing, hunting, trapping, and oral immunization were implemented to control the CSF infection spread [3]. The effects of those measures have been evaluated using surveillance data. With regard to fencing, it could be a method to restrict wild boar movement and to prevent the spread of CSF virus (CSFV), but the efficacy of fencing depends on the intactness of the fences and the practical feasibility in larger areas is limited [3]. Hunting and trapping are methods to reduce the number of susceptible wild boars. Theoretically, it is considered necessary to achive the reduction of more than 70–80% of the population to reduce CSFV spread, and this goal is rarely reached in practice. Hunting and trapping method alone are not considered to be efficient for CSF control, though they can be useful as complementary control measures and necessary for sampling [3, 10, 11]. Oral vaccination has proved to be effective in maintaining herd immunity and achieving CSF control and is considered to be the only available method for CSF eradication in large forested areas [3, 12, 13]. Moreover, it has been recommended that control measures should consider the geographical conditions, as well as the social structure, spatial distribution, and density of the affected wild boar population [3, 10, 11].

**Fig. 1:**
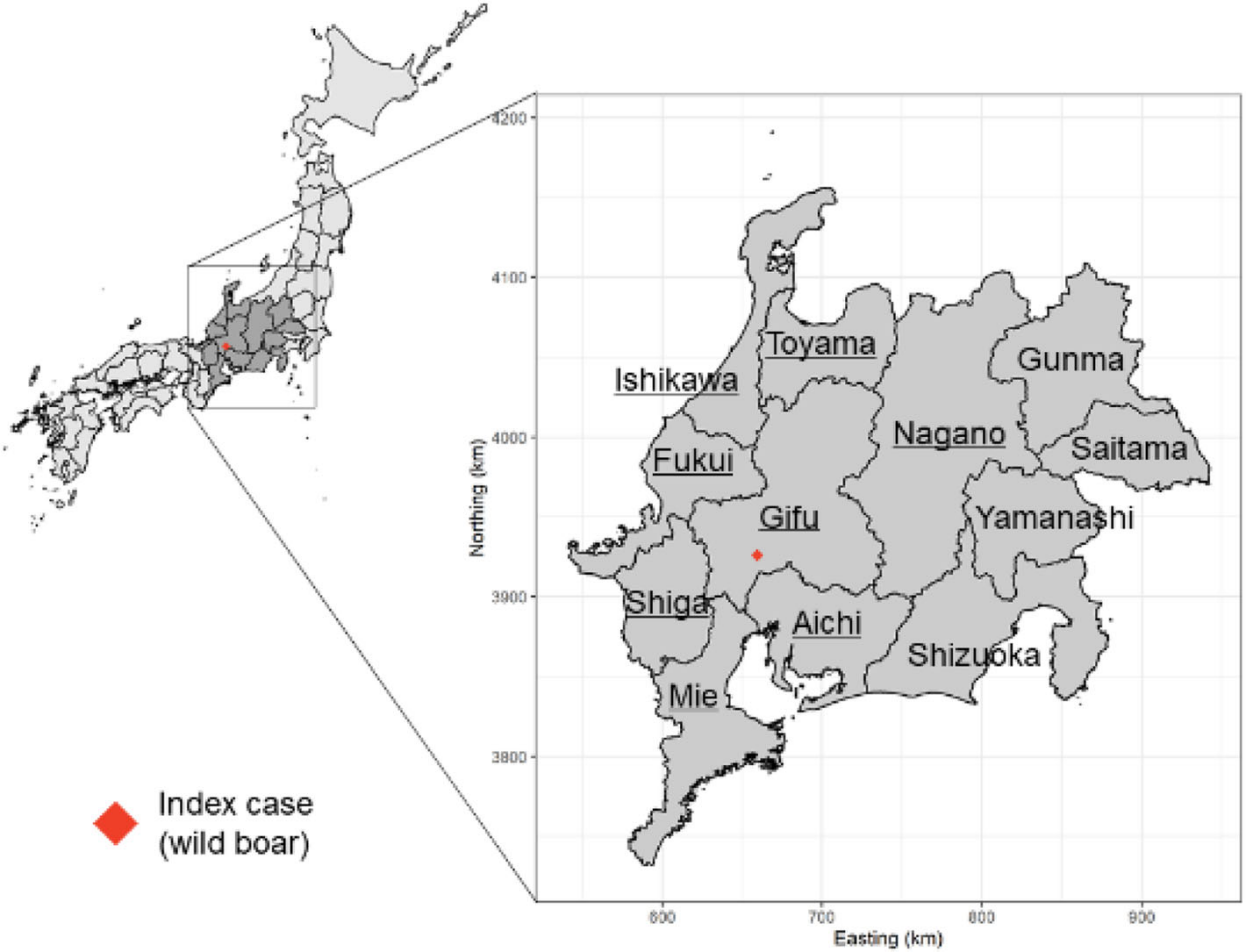
Location of the index case of a CSF-infected wild boar in Japan and the study area. Prefectures (8) with the underlined names comprise the area included in the analysis of the CSF spread velocity

**Fig. 2:**
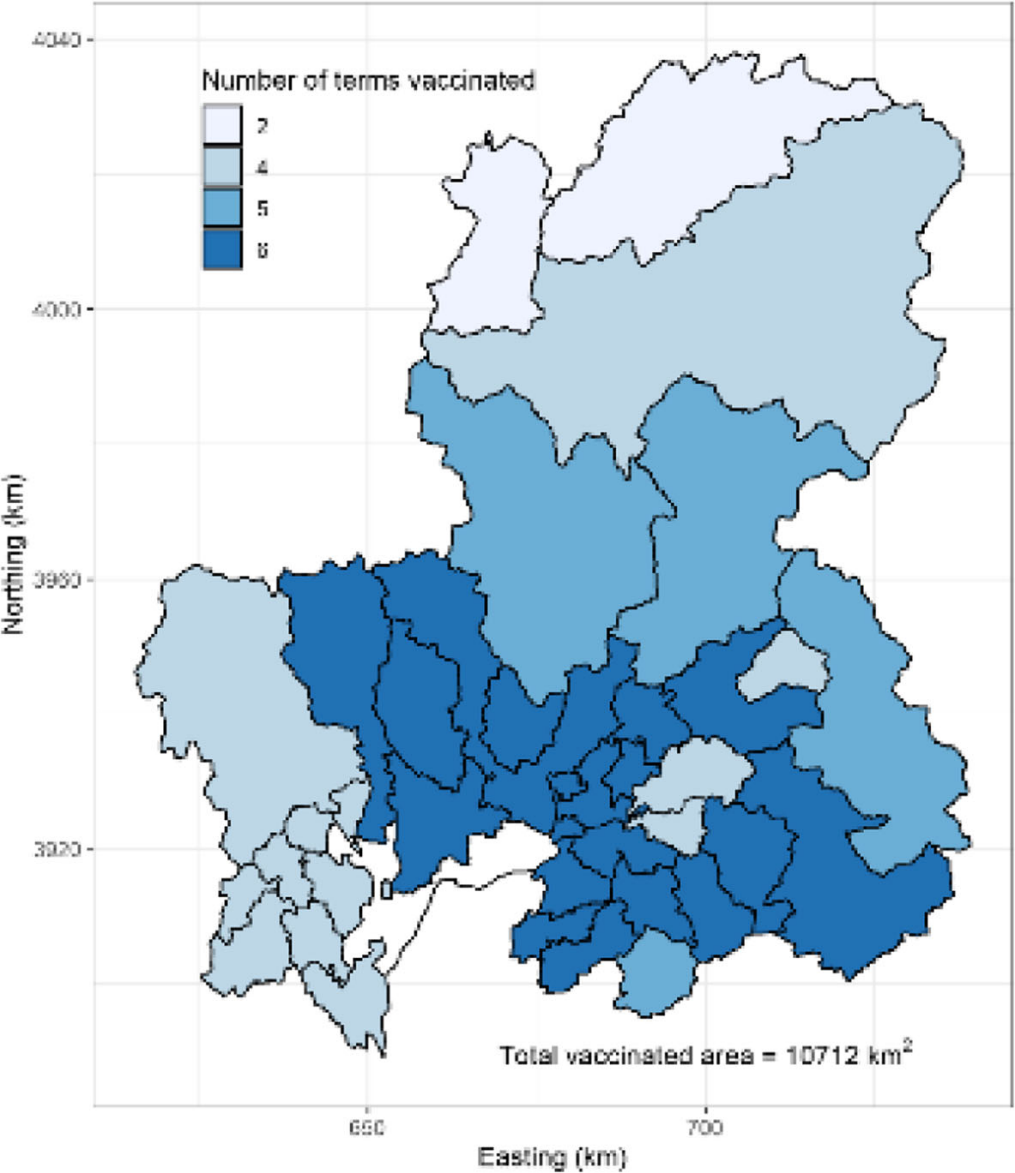
The vaccinated areas of the Gifu and northern Aichi prefectures from March 2019 to February 2020. The number of terms vaccinated ranged from 0 to 6 in each municipality

In Japan, CSF re-emerged in 2018 after 26 years of absence [6, 14, 15]; specifically, shortly after the first case was reported at a pig farm, a dead wild boar with CSF infection was found at a 7.4 km distance from the index farm. This was the first case of the CSF epidemic in wild boars in Japan. Subsequent CSF surveillance in dead or captured wild boars revealed that the infection had spread widely in the local wild boar population [16, 17]. Consequently, several control measures for wild boars were implemented. Initially, intensive capturing for surveillance and depopulation started in September 2018. The target areas covered a 10-km radius from affected farms or locations of infected wild boars. Captured wild boars underwent virological and/or serological testing by prefectural veterinary services. The target areas of active investigation were gradually expanded according to the disease spread among pigs and wild boars. Next, fencing began in October 2018 in the Gifu Prefecture, in the surrounding area of the location of infected wild boars to prevent wild boar movement to outer areas. Moreover, fences were placed in new detection sites of infected wild boars and extended several times until March 2019. Finally, oral immunization was first started in March 2019 in parts of the Gifu Prefecture and the neighboring Aichi Prefecture (Figs. 1 and 2). The areas to be vaccinated were expanded along with the spread of CSF in wild boars (Supplementary Fig. 1). The bait vaccines were imported from Germany by the Ministry of Agriculture, Forestry, and Fisheries (MAFF) of Japan and provided to designated prefectures.

Since infected wild boars pose a major infection risk to pig farms [18], there is a need to control CSF spread in wild boars to prevent its introduction to pig farms. An artificial infection study revealed that the recent Japanese isolate of the CSFV in wild boars was moderately virulent; moreover, the effectiveness of the bait vaccine was confirmed through an experimental CSFV challenge using boar-pig hybrids as a wild boar alternative [19]. However, characteristics of the affected wild boar population and the field effectiveness of the oral bait vaccination remained unclear. Consequently, we aimed to analyze the surveillance data of wild boars until a year after vaccination commencement.

## Results

### Descriptive analysis of CSF spread in wild boars

During the study period, 13,216 wild boars were found dead or captured; among them, 12,669 animals underwent polymerase chain reaction (PCR) tests. Among them, data of 12,306 animals were available with individual information (notification date, capture method [found dead or captured], sex [male or female], age [adult or piglet]); moreover, data of 11,516 animals were available with locational information regarding the latitude and longitude.

Among the 12,306 animals, 1145 (9.3%) were found dead; 11,161 (90.7%) were captured; 5987 (48.7%) were females; 6319 (51.3%) were males; 8873 (72.1%) were adults; and 3433 (27.9%) were piglets. There was no significant sex difference in the percentage of dead animals (9–10%; chi-squared test, *p* > 0.05). However, there was a significant difference in the percentage of dead animals between adults (7.1%) and piglets (15%) (chi-squared test, *p* <  0.001) (Table 1).

**Table 1:** Summary of tested wild boars with individual information

Characteristic	Overall *N* = 12,306	Found dead *N* = 1145 (9.3%)^a^	Captured *N* = 11,161 (91%)^a^	*p*-value^b^
Sex				0.077
Male	6319	559 (8.8%)	5760 (91%)	
Female	5987	586 (9.8%)	5401 (90%)	
Age				< 0.001
Piglet	3433	516 (15%)	2917 (85%)	
Adult	8873	629 (7.1%)	8244 (93%)	

^a^Statistics presented: n (%)

^b^Statistical tests performed: chi-square test of independence

**Fig. 3:**
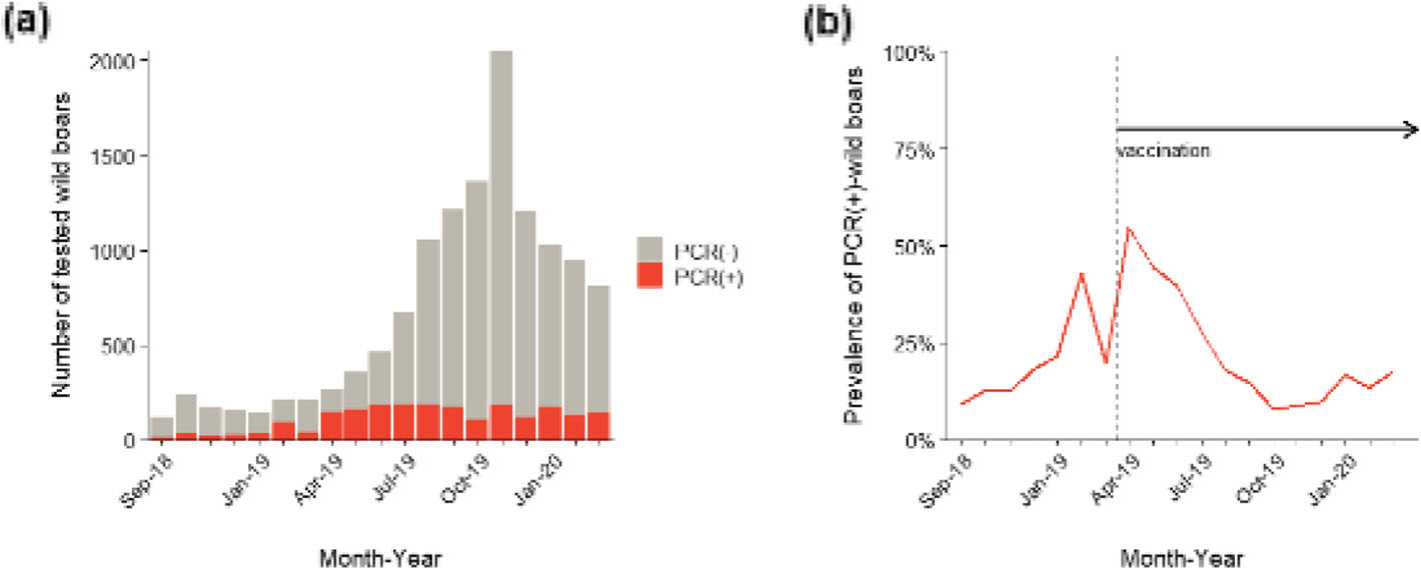
Temporal trends of CSF infection in wild boars from September 2018 to March 2020. **a** Absolute monthly numbers of CSF-tested wild boars. PCR-positive wild boars were constantly found dead or captured. **b** Prevalence of PCR-positive wild boars (red solid line)

Figure 3 presents the temporal trend of CSF spread, which was derived from the 12,669 animals with available PCR test results. The percentage of PCR-positive animals increased after September 2018. Although there was a fluctuation before and after oral vaccine implementation (March and April in 2019), the percentage of PCR-positive animals gradually decreased to 8% in October 2019 and remained at 10–20% after that. The major reason for the sudden drop may be the prohibition of hunting from that period; i.e. shortly before the start of the distribution of bait vaccines, to assure the consumption of bait vaccines.

**Fig. 4:**
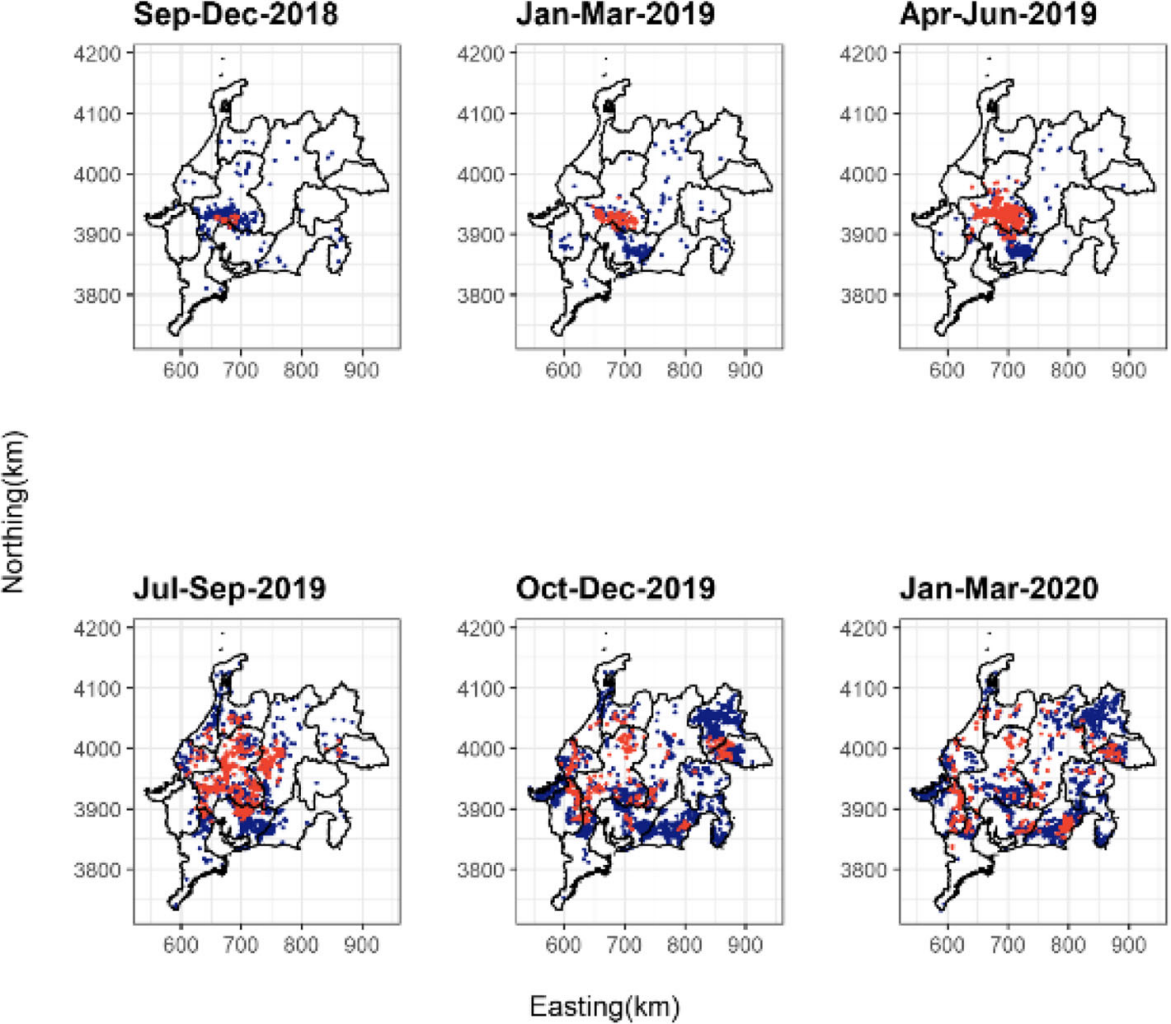
Spatial spread of CSF-infection in wild boars according to 3-month or 4-month terms. Red and blue dots indicate PCR-positive and PCR-negative wild boars, respectively (one dot represents one individual)

Figure 4 presents the geographic spread of CSF infection, which was derived from the 11,516 animals with available locational information. In the early outbreak phase (between September 2018 and March 2019), PCR-positive animals were only detected in the southern part of the Gifu Prefecture and the northern part of the Aichi Prefecture. However, from April to September 2019, the infection widely spread to the northern and eastern areas followed by the southern and western areas.

**Fig. 5:**
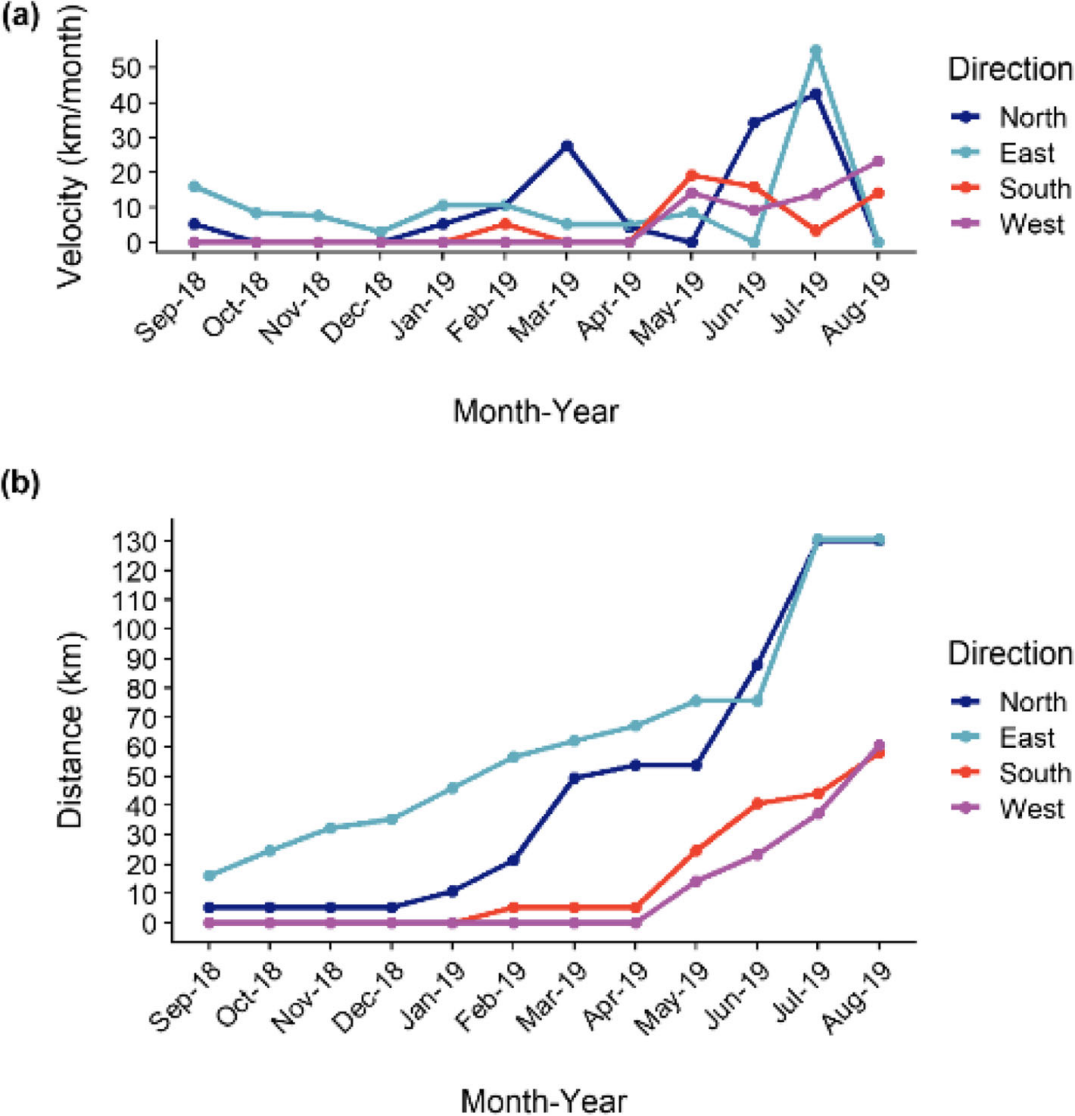
CSF spread in wild boars in different direction from the location of the index case. **a** Monthly spread velocity. **b** Cumulative distance from the index case

The average CSF spread velocity in the north/east, south, and west was 10.9 km/month, 4.8 km/month, and 5.0 km/month, respectively. From April to July 2019, the CSF infection spread especially to the north and east, with the velocities for both directions peaking in July 2019 (42.4 km/month and 54.9 km/month, respectively) (Fig. 5a). Contrastingly, the spread velocities to the south (19.3 km/month) and west (23.3 km/month) peaked in May and August 2019, respectively. The total spread distances until August 2019 were about 130 km to the north and east and about 60 km to the south and west. (Fig. 5b).

### Factors associated with CSF infection

Table 2 summarizes the data of the 1824 animals used for the pre-vaccination statistical model analysis. There was no strong correlation between any of the variables (Cramer’s V ≤ 0.2). The generalized linear mixed model (GLMM) analysis was conducted using all variables except “sex”, which had a *p*-value > 0.2 in the univariate analysis. The final model selected based on the Akaike’s Information Criterion (AIC) values comprised two variables (“captured method” and “age”), without interaction terms. Table 3 presents the estimated effects of each variable. Regarding the “capture method”, the infection probability in dead animals was significantly higher than that in captured animals (Estimated odds ratio (OR) of 6.15, 95% confidence interval (CI): 3.54–10.7). Moreover, the infection probability was significantly higher in adults than in piglets (OR of 2.14, 95% CI: 1.49–3.09).

**Table 2:** Summary of explanatory variables included in the generalized linear mixed model for pre-vaccination data

Variables	Infected *N* = 208 (11%)^a^	Susceptible *N* = 1616 (89%)^a^	*p*-value^b^
Capture method			< 0.001
Captured	167 (9.6%)	1571 (90%)	
Dead	41 (48%)	45 (52%)	
Sex			0.8
Male	103 (11%)	820 (89%)	
Female	105 (12%)	796 (88%)	
Age			0.049
Piglet	72 (9.6%)	679 (90%)	
Adult	136 (13%)	937 (87%)	
Season			< 0.001
Spring (Mar–May)	27 (20%)	110 (80%)	
Summer (Jun–Aug)	6 (2.5%)	231 (97%)	
Autumn (Sep–Nov)	76 (8.3%)	840 (92%)	
Winter (Dec–Feb)	99 (19%)	435 (81%)	

^a^Number and proportion of animals with PCR test results

^b^Statistical tests performed: chi-square test of independence

**Table 3:** Effect of variables estimated by the selected binomial generalized linear mixed model for pre-vaccination data

Variables	OR^a^	95% CI^a^	*p*-value
Capture method			
Captured	–	–	
Dead	6.15	3.54, 10.7	**< 0.001**
Age			
Piglet	–	–	
Adult	2.14	1.49, 3.09	**< 0.001**

*p*-values < 0.05 are marked in bold letters

^a^*OR* Odds Ratio, *CI* Confidence Interval

Table 4 summarizes the data of the 3684 animals used for the post-vaccination statistical model analysis. There was no strong correlation between any of the variables (Cramer’s V ≤ 0.2). The GLMM analysis was conducted using all variables except “sex”, which had a *p*-value > 0.2 in the univariate analysis. The final model selected based on AIC values comprised three variables (“capture method”, “age”, and “season”) and two interaction terms (“capture method: age” and “capture method: season”). The models including the variable “vaccination” were not selected as the final model. Table 5 presents the effects of each variable estimated by this model. In contrast to the pre-vaccination model, there were no differences in infection probability between dead and captured animals. However, the infection probability was significantly higher in adults than in piglets (OR of 2.08, 95% CI: 1.67–2.61) as in the pre-vaccination model, and in adult animals that were found dead (OR of 3.98, 95% CI: 1.58–10.1, interaction term) than in those that were captured. Regarding the season, the infection probability was lower in autumn than in spring (OR of 0.52, 95% CI: 0.33–0.81), but higher in dead animals that were found in seasons other than spring (OR of 3.43, 95% CI: 1.17–10.0, for dead animals in summer; OR of 8.81, 95% CI: 2.88–27.0, for dead animals in autumn; OR of 6.58, 95% CI: 1.62–26.8, for dead animals in winter).

**Table 4:** Summary of explanatory variables included in the generalized linear mixed model for post-vaccination data

Variables	Infected *N* = 1076 (29%)^a^	Susceptible *N* = 2608 (71%)^a^	*p*-value^c^
Capture method			< 0.001
Captured	876 (25%)	2574 (75%)	
Dead	200 (85%)	34 (15%)	
Sex			0.7
Male	541 (29%)	1331 (71%)	
Female	535 (30%)	1277 (70%)	
Age			0.2
Piglet	336 (31%)	761 (69%)	
Adult	740 (29%)	1847 (71%)	
Season			< 0.001
Spring (Mar–May)	301 (60%)	201 (40%)	
Summer (Jun–Aug)	318 (53%)	282 (47%)	
Autumn (Sep–Nov)	271 (16%)	1392 (84%)	
Winter (Dec–Feb)	186 (20%)	733 (80%)	
Vaccination^b^			0.007
Vac_0	829 (28%)	2113 (72%)	
Vac_1	247 (33%)	495 (67%)	

^a^ Number and proportion of animals with PCR test results

^b^Vac_0: after 1 to 2 vaccination terms; Vac_1: after 3 to 6 vaccination terms

^c^Statistical tests performed: chi-square test of independence

**Table 5:** Effect of variables estimated by the selected binomial generalized linear mixed model for post-vaccination data

Variables	OR^a^	95% CI^a^	*p*-value
Capture method			
Captured	–	–	
Dead	0.99	0.42, 2.30	0.97
Age			
Piglet	–	–	
Adult	2.08	1.67, 2.61	**< 0.001**
Season			
Spring (Mar–May)	–	–	
Summer (Jun–Aug)	0.79	0.50, 1.24	0.30
Autumn (Sep–Nov)	0.52	0.33, 0.81	**0.004**
Winter (Dec–Feb)	0.82	0.57, 1.18	0.28
Capture method * Age			
Dead * Adult	3.98	1.58, 10.1	**0.004**
Capture method * Season			
Dead * Summer (Jun–Aug)	3.43	1.17, 10.0	**0.025**
Dead * Autumn (Sep–Nov)	8.81	2.88, 27.0	**< 0.001**
Dead * Winter (Dec–Feb)	6.58	1.62, 26.8	**0.008**

*p*-values < 0.05 are marked in bold letters

^a^*OR* Odds Ratio, *CI* Confidence Interval

### Pre- and post-vaccination infection status of the wild boars

**Fig. 6:**
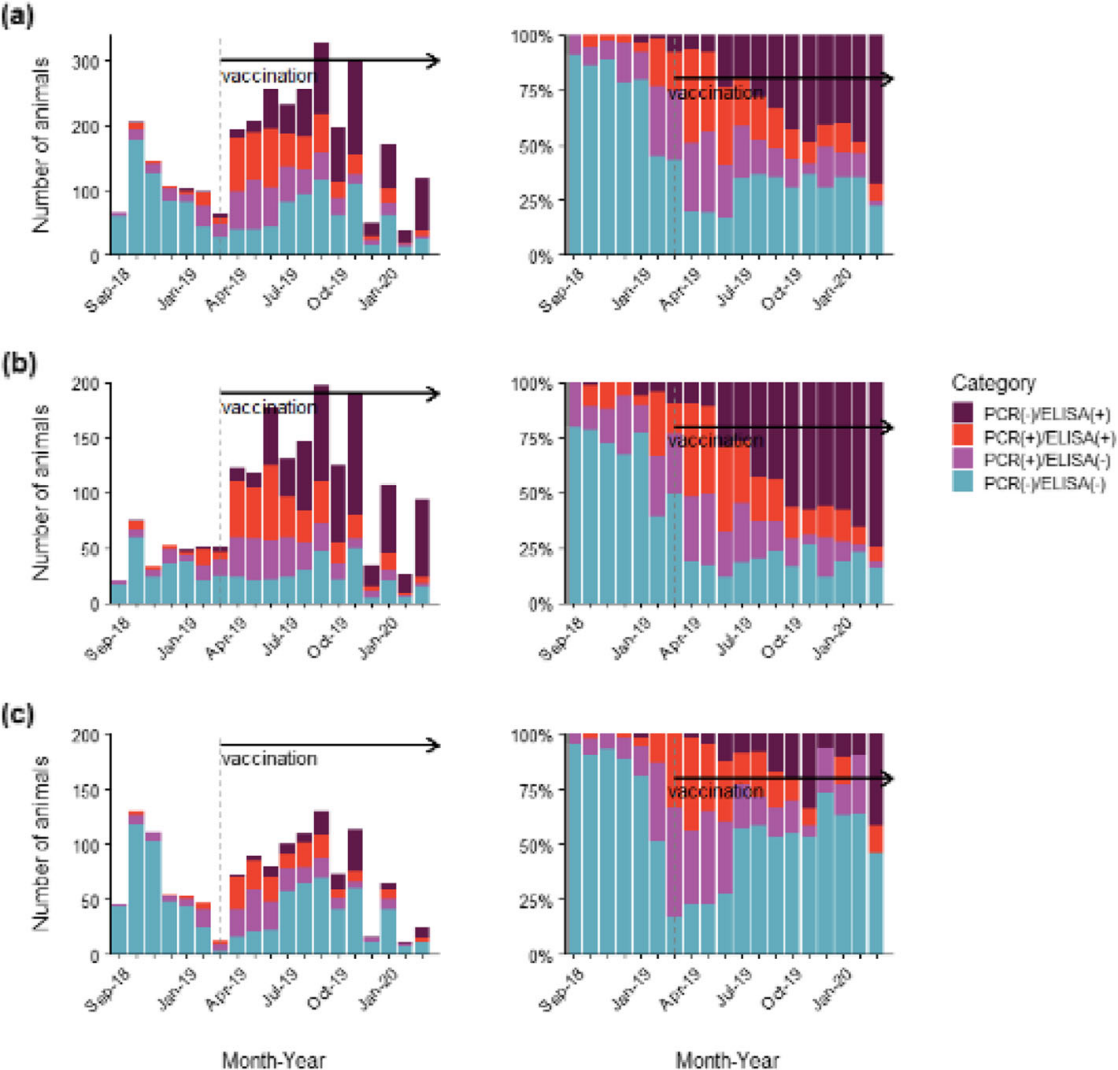
Immunization through oral bait vaccination in Gifu and northern Aichi. **a** All wild boars, **b** Adult wild boars, **c** Piglets. Left: Absolute monthly numbers of the tested wild boars. Right: Monthly proportion of each category

Figure 6 presents the infection status categorized using the results of PCR and enzyme-linked immunosorbent assay (ELISA) tests in the Gifu and northern Aichi prefectures.

The proportion of susceptible animals, i.e. PCR(−)/ELISA(−) animals, decreased from 49 to 16% in adults at 1 year post vaccination. In piglets, the proportion increased from 17 to 73% in December 2019, with a subsequent decrease; however, it was 46% after 1 year post vaccination.

The pre-vaccination proportion of infected animals, including PCR(+)/ELISA(−) and PCR(+)/ELISA(+), was 57 and 49% in adults and piglets, respectively. After 1 year of vaccination, the proportion decreased to 10% in adults and 13% in piglets. Among the infected animals, the percentage of adults with antibodies, i.e. PCR(+)/ELISA(+) animals, before and 1 year after vaccination was 29 and 6%, respectively. The proportion of infected piglets with antibodies was 13% before vaccination; subsequently, it increased to 42% in April 2019 and decreased to 13% after 1 year of vaccination.

Immune animals, i.e. PCR(−)/ELISA(+) animals, were identified before and after vaccination. The pre-vaccination proportion of immune adults and piglets was 4 and 0%, respectively. After 1 year of vaccination, these proportions increased to 74% in adults and 42% in piglets.

## Discussion

This study used wild boar surveillance data from September 2018 to March 2020 to descriptively analyze the characteristics of the CSF epidemic in wild boars. There was a faster infection spread in the northern and eastern areas than in the southern and western areas. Regarding the CSF infection in wild boars, the capture method and age were positively associated with infection while the influence of the number of vaccination terms was not elucidated by the statistical modeling analyses. After oral bait vaccine distribution began in March 2019, there was a decrease and increase in the proportion of infected and immune animals, respectively.

### Spatial and temporal characteristics of CSF spread in wild boars

The spatial spread of infection was geographically continuous from the location of the first detected case in September 2018 until August 2019. During this period, the CSF spread velocity among wild boars was approximately 5–10 km/month (15–30 km/quarter year) in Japan. Previous studies on the CSF spread velocity in wild boars in Germany and France reported average spread velocities of 7.6–15.6 km/quarter year [12, 20]. These results indicate that the CSF spread velocity in Japan was faster than that in Europe.

One of the most important factors affecting the CSF spread in wild boars is the forest coverage, considering forest being the major habitat of wild boars [13]. Previous studies in France have shown that CSF infection in wild boars spread more likely in populations inhabiting large continuous forest areas [21, 22]. In contrast to central and western European countries, where the forest coverage (forest area compared to the percentage of total land area) is almost ≤30% [23] and forestlands tend to be fragmented and independent [24], forest coverage in Japan is more than 66% nationwide and more than 81% in the Gifu Prefecture [23, 25]. In addition, compared to Europe, Japan has a considerably higher number and population density of wild boars [26–30]. Consequently, the rich forest environment and higher population density could have resulted in the higher spread velocity and rampant spread of CSF in wild boars in Japan.

Additionally, we observed faster CSF spread to the north and east than to the south and west. This could be attributed to the continuity of the forest land in the north and east, while urban areas extended to the south of the location of the first reported case. Moreover, there was a relatively higher proportion of agricultural land to the west (Supplementary Fig. 2). However, there is also a possibility that the observed velocity was affected by direction-based differences in the surveillance intensity. Given that the first case was located at the southern end of the forest area in the Gifu Prefecture, with subsequent cases in the eastern and northern areas in the early epidemic phase, there was greater surveillance on captured wild boars in the northern and eastern areas. Consequently, surveillance in the south and west directions was lower, which could have led to some infected wild boars being overlooked.

### Risk factors associated with CSF infection

We found that animals found dead had a significantly higher infection probability than captured animals in the pre-vaccination period. Although the animals could have died due to reasons other than CSF infection and the higher percentage of dead piglets might be caused by other pathogens, the results of GLMM analyses indicated that the possibility of CSF infection was higher in the animals that were found dead. This finding suggests that CSF infection resulted in higher mortality among wild boars and is consistent with a previous report regarding a CSF epidemic among wild boars in France [21].

We found that compared with piglets, there was a significantly higher pre- and post- vaccination proportion of infected adult animals. However, previous studies in Germany and France reported a higher CSF infection risk or incidence in young animals than in subadult and adult animals [20, 21]. These inconsistent findings between Japan and Europe could be attributed to several factors. First, it could be attributed to the higher fatality in CSF-infected piglets [31] than in adults, under the condition of the dense forest coverage in Japan. This could lead to CSF-infected piglets dying unnoticed in forest areas without easy human access. Moreover, there are differences in the surveillance method between Japan and Europe. In Europe, the main hunting method is gun shooting; moreover, young animals are preferentially targetted since they are difficult to be protected using oral vaccines [11]. Contrastingly, in Japan, the main capturing method is trapping (mainly using loop and box traps), without specifically targeted age groups, in areas approachable by hunters; therefore, capturing CSF-infected and weakened piglets might be difficult. Given the aforementioned factors, the proportion of infected piglets in Japan might have been underestimated.

Seasonal effects on CSF infection in wild boars appeared to differ before and after vaccination. The dead animals other than in spring indicated significantly higher probability of infection in the post-vaccination period. However, the one-year study period may be insufficient for analyzing the seasonal effects on infection, and there is a need for further research using data obtained across several seasons.

Regarding vaccination, although we had expected a positive effect of more than two terms of vaccine distribution in reducing infection, the influence was not elucidated by our study. This result might indicate that the vaccination implemented during the study period did not significantly reduce the risk of infection in the overall wild boar population.

### Effect of oral vaccination and future challenges in CSF control in Japan

After the 1-year oral vaccination program (maximum distribution: six times) in Gifu and northern Aichi areas, there was an apparent increase in the proportion of immune animals, which reached approximately 80% in adults and 50% in piglets. Regarding CSF outbreaks in Europe in the early 2000s, immunization of more than 60% of animals was estimated to help achieve CSF eradication in the wild boar population [10]. However, while the prevalence after vaccine distribution in Germany and France was less than 1% [12, 32], 10% of animals in Japan remained infected after 1 year of vaccine distribution. Given the higher prevalence in Japan and lower pathogenicity of the causative strain [19], there would be a need for greater immunization levels to allow CSF eradication in the wild boar population in Japan.

There are differences in CSF control in wild boars between Europe and Japan. First, as aforementioned, there is a higher forest coverage in Japan; moreover, forestlands tend to be spread with continuity, and there are a higher number and population density of wild boars. Additionally, more than 70% of the forest area in France and Germany is rather flat (with 0–15° slope); contrastingly, more than 50% of the forest area in Japan is rather steep (with more than 15° slope) [33]. Therefore, manual vaccine distribution targeting wild boars could be more difficult in Japan. To overcome this difficulty, helicopters are used for oral vaccine distribution in several Japanese areas. Nonetheless, the area coverage remains limited given the very high implementation cost. Since CSF tends to persist in a large and dense wild boar population [9, 22, 34], compared with Europe, Japan might show more persistent CSF infection in its wild boar population. Further, the lack of effective juvenile-targeted hunting in Japan led to the proportion of susceptible piglets remaining high after 1 year of vaccination. Generally, wild boar populations are comprised of more juveniles than adults; moreover, the reported ratio of juveniles to adults is 3–4 to 1 [35]. However, the ratio of juveniles to adults in our study was 0.4 to 1; therefore, there may be a higher proportion of susceptible animals in the entire wild boar population given the observed local boar population in our study.

The aforementioned findings indicate the need to address the increased infection in piglets and sub-adult animals, as well as the associated infection persistence and spread, to achieve CSF eradication in the wild boar population in Japan. Continuous surveillance to monitor the infection status in the wild boar population could yield a better control strategy for CSF infection in the wild boar population in Japan.

## Conclusion

Our findings suggest that CSF infection in the wild boar population spreads faster in Japan than in European countries and that the immunization of young wild boars after 1 year of oral bait vaccination is insufficient. Conditions associated with wild boars’ habitat, including forest continuity, higher wild boar population density, and a larger proportion of susceptible piglets, would impede the efficacy of control measures against CSF infection in wild boars. Accordingly, the improvement of national control strategy against the CSF epidemic in wild boars based on the continuous surveillance of wild boars will be necessary to achieve the eradication of CSF in Japan.

## Methods

### Overview of wild boar surveillance

Wild boar surveillance has been implemented in cooperation with the hunting associations of each prefecture. The major hunting methods in Japan use box and loop traps. Among the wild boars captured and tested from September 2018 to March 2020, more than 80 and 5% of animals were hunted by traps and shooting, respectively.

Since the re-emergence of CSF, there has been countrywide surveillance of dead wild boars (including in non-affected prefectures). In affected prefectures, the intensive capturing areas were within a 10-km radius from affected farms and locations where infected wild boars were found dead or captured. Moreover, in neighboring prefectures, there was intensive wild boar capturing around pig farms and at the borders.

Additionally, in the area conducting oral bait vaccination, there was intensive capturing in the mountains, forests, and wood edges for monitoring the immunization status. Wild boar capturing for monitoring was implemented from 10 days of vaccine distribution to reduce the possibility of detecting viral genes derived from the vaccine strain.

As the target areas for wild boar surveillance were expanded along with the expansion of the CSF-affected areas, this study focused on the analyses of the situation of CSF infection of wild boars in the CSF-affected areas by using the surveillance data. This approach resulted in minimizing the influence of the condition of wild boars that inhabited the areas without the risk of exposure to CSFV.

Samples obtained from dead or captured wild boars were tested by each prefectural veterinary service. For dead animals, the tonsils, spleen, or kidneys were sampled and used in PCR for virus detection. Further, when blood could be collected, serum samples were used for PCR and the ELISA antibody detection assay. For captured animals, serum samples were obtained, and PCR and ELISA assays were performed. The PCR assay used for detection of CSFV was the reverse transcription PCR (RT-PCR) assay to amplify the 5′-unstranslated region (UTR) of CSFV using primers 324 (5′-ATG CCC (T/A) TA GGA CTA GCA-3′) and 326 (5′-TCA ACT CCA TGT GCC ATG TAC-3′) based on Vilcek et al. [36] For ELISA assay, the Classical Swine Fever ELISA kit II (JNC Corp., Tokyo, Japan) was used.

When samples were obtained from wild boars found dead or captured after vaccination in vaccinated areas, specifically at 11–15 post-distribution days and within 2-km of the distribution points, and were confirmed as PCR-positive, the samples were considered to be influenced by vaccination, and genomic sequencing for differentiating between the vaccine and field strain was performed at the National Institute of Animal Health, National Agriculture and Food Research Organization. We excluded samples containing the vaccine strain genome from the PCR-positive data.

### Overview of oral vaccination

For oral bait vaccination, the commercial bait vaccine (Pestiporc Oral, IDT Biologika GmbH, Dessau-Rosslau, Germany), an attenuated CSF vaccine, was used. Prefectures that implemented oral bait vaccination were designated by the MAFF based on areas where infected wild boars were found. Vaccine distribution began late in March 2019 in some municipalities of the Gifu and Aichi Prefectures (Figs. 1 and 2). Based on the expansion of areas with CSF-positive wild boars, bait vaccination was started in prefectures neighboring the Gifu Prefecture as follows: from July 2019 (Mie, Fukui, and Nagano) and from August 2019 (Toyama and Ishikawa) (Supplementary Fig. 1). After September 2019, vaccination began in other prefectures, including Shiga, Shizuoka, and Gunma, that surrounded habitats with CSF-positive wild boars (Supplementary Fig. 1). Vaccine distribution began with double vaccination thrice a year; specifically, during spring, summer, and winter [13]. The locations for vaccine distribution in each municipality were selected for each prefecture based on the wild boar habitats and surrounding environments.

### Study period, study area, and data collection

The study period was between September 2018, when the first CSFV-positive wild boar was reported, to March 2020, which marked 1 year after starting oral bait vaccination. The study area was designated as the 12 prefectures where CSFV-positive wild boars were found during the study period (Fig. 1).

In this study, data regarding wild boar surveillance included CSF results obtained through PCR and/or ELISA conducted at prefectural veterinary services, as well as the individual information (notification date, capture method [found dead or captured], sex [male or female], age [adult or piglet], and location [latitude and longitude]). Data were reported from prefectural governments to the MAFF and provided for analysis in this study.

In this study, other than in the statistical modeling analyses, CSF-infected animals were defined as those with positive PCR results for the virus. We excluded six samples with PCR-positive results that were found to express the vaccine strain genome.

In the statistical modeling analyses, for pre-vaccination data, the animals with immune [PCR(−)/ELISA(+)] status were also considered as CSF-infected. Animals with [PCR(−)/ELISA(+)] status were removed from the analysis for post-vaccination data because discrimination of infected from vaccinated but not infected animals was not possible in these animals.

### Velocity of spread

We investigated the CSF spread velocity in the wild boar population by including the major affected areas, including the Gifu Prefecture and its seven adjacent prefectures (shown in Fig. 1). The analysis period was from September 2018 to August 2019. Among the infected animals reported during this period, 1125 animals with available data regarding the latitude and longitude were used to calculate the spread velocity.

Detecting infected animals is indicative of the infection status of the area where the animals are found. Therefore, instead of using point data of the infected animals, grid data of the area, which was divided into hexagonal grids (area: 25 km^2^; center-to-center distance: ≈ 5.4 km), was used to calculate the CSF spread velocity. A grid was considered infected if an infected wild boar was detected in it; moreover, its infection date was defined as the date when the first infected animal was detected in it. The first-infected grid referred to the grid that had the first infected wild boar. First, we calculated the Euclidean distances between the centers of each infected grid and the first-infected grid; subsequently, we classified them into the four cardinal directions based on the azimuthal angles from the first-infected grid. Next, the maximum spread distances per month for each direction, as well as the differences (km) between the maximum distance of each month and that of the previous month were determined. The latter value was considered as the monthly velocity of infection spread (km/month).

### Statistical modeling analyses

Factors associated with CSF infection were analyzed using a statistical model. Data obtained from animals with available PCR and ELISA test results and individual information were used for the analysis. To evaluate the vaccination influence, data were classified as pre- and post-vaccination as follows: (i) pre-vaccination: animals were found dead/captured in prefectures where oral vaccination had not begun and (ii) post-vaccination: animals were found dead/captured in municipalities where oral vaccination had begun. Based on this classification, we included pre- and post-vaccination data from 1824 and 3684 animals, respectively.

First, univariate analysis was conducted on each pre- and post-vaccination dataset. The objective variable was the infection status of each animal. For pre-vaccination data, the objective variable was classified as infected [PCR(+)/ELISA(−), PCR(+)/ELISA(+) and PCR(−)/ELISA(+)] or susceptible [PCR(−)/ELISA(−)], and for post-vaccination data, the objective variable was classified as infected [PCR(+)/ELISA(−) and PCR(+)/ELISA(+)] or susceptible [PCR(−)/ELISA(−)]. The explanatory variables were the “capture method” (found dead or captured), “sex” (female or male), “age” (adults or piglets), and “season” (detected seasons). Seasons were classified based on the reporting date as follows: March–May, spring; June–August, summer; September–November, autumn; and December–February, winter. Regarding post-vaccination data, we conducted a univariate analysis with the vaccination status as an explanatory variable, which was dichotomized based on the number of vaccinated terms of each municipality as of each animal’s reporting date. Based on the median number of vaccinated terms (i.e., two terms), the vaccination status was categorized as having ≤2 or ≥ 3 vaccination terms. All univariate analyses were conducted using the chi-squared test; moreover, subsequent multivariate analysis was conducted using explanatory variables with *p*-values < 0.2 at univariate analyses. The between-variable correlation was determined by calculating Cramer’s V.

Multivariate analysis was conducted using GLMM with random effects. The study period was divided into six periods (period 1: September–December 2018, period 2: January–March 2019, and subsequently at 3-month intervals); further, the periods and prefectures were added as random effects. Modeling was conducted using the glmer function (with a binomial distribution and log link function) of the lme4 package of R [37]. For explanatory variables other than the vaccination status, interaction terms of variables were also considered in the modeling. The model was selected using the dredge function of the MuMIn package [38], following AIC-based ranking. The model with convergence and the least number of explanatory variables was selected as the final model. All statistical analyses were conducted using R version 4.0.2 (R Core Team, 2020, R: A language and environment for statistical computing. R Foundation for Statistical Computing, Vienna, Austria. URL https://www.R-project.org/.).

### Immunization after oral bait vaccination

For the analysis of immunization after oral bait vaccination, the vaccinated areas in Gifu and the northern area of Aichi prefecture (Fig. 2) were targeted in this study, where oral vaccination was first implemented in Japan and where vaccines were distributed for a maximum of six terms. Table 6 presents the number of distributed vaccines and the size of the vaccinated area in each term. We included 3131 animals tested in the vaccinated area during the study period with available information regarding PCR/ELISA tests and age. The infection status of wild boar (susceptible [PCR(−)/ELISA(−)], infected [PCR(+)/ELISA(−) and PCR(+)/ELISA(+)], and immune [PCR(−)/ELISA(+)]) [20, 31] was examined in adults and piglets, respectively. Each status was interpreted as follows:

- Susceptible: animals that may become infected.
- Infected: animals that are infected with the wild CSFV strain.
- Immune: animals that have recovered from natural infection with wild CSFV strain or have been immunized through vaccination.

**Table 6:** Oral bait vaccination at the Gifu and northern Aichi prefectures

Term	Vaccination period (month/year)	Vaccinated area (km^2^)	Number of baits distributed	Distribution density (baits/km^2^)
1–1	03–04/2019	3255	26,401	8.1
1–2	04–05/2019	5924	30,630	5.2
2–1	07/2019	9378	39,520	4.2
2–2	08/2019	10,527	44,770	4.3
3–1	12/2019	10,712	45,280	4.2
3–2	02/2020	9563	37,900	4.0

### Geographical information

Geographical data regarding the administrative divisions (as of 2018), forested areas (as of 2015), agricultural areas (as of 2015), and urban areas (as of 2011) were downloaded from the National Land Numerical Information download service, which is provided by the Ministry of Land, Infrastructure, Transport, and Tourism of Japan, and used for map drawing. The maps were drawn using R and QGIS (QGIS.org, 2021. QGIS version 3.10. Geographic Information System. QGIS Association. http://www.qgis.org).

## Supplementary Information

**Additional file 1: Supplementary Fig. 1.** Distribution of oral bait vaccines. The prefectures where vaccines were distributed were gradually expanded according to the CSF spread in the areas.

**Additional file 2: Supplementary Fig. 2.** Geographic information on the area surrounding the index case of a CSF-infected wild boar. Dots indicate the location and PCR test results of wild boars found dead or captured from September 2018 to August 2019 (the period for spread velocity analysis).

## Data Availability

The datasets used and/or analyzed during the current study are available from the corresponding author on reasonable request.

## Abbreviations

CSF: Classical swine fever
CSFV: Classical swine fever virus
CI: Confidence interval
ELISA: Enzyme-linked immunosorbent assay
GLMM: Generalized linear mixed model
MAFF: Ministry of Agriculture, Forestry and Fisheries
OR: Odds ratio
PCR: Polymerase chain reaction
AIC: Akaike’s Information Criterion
