# Supplementary figures and images for: Epidemiological analysis of classical swine fever in wild boars in Japan
Source: BMC Vet Res. 2021 May 11;17:188. doi: 10.1186/s12917-021-02891-0 (PMC8111369; doi:10.1186/s12917-021-02891-0)

Start of vaccine distribution

- March 2019
- July 2019
- August 2019
- September 2019
- October 2019
- November 2019
- December 2019–March 2020

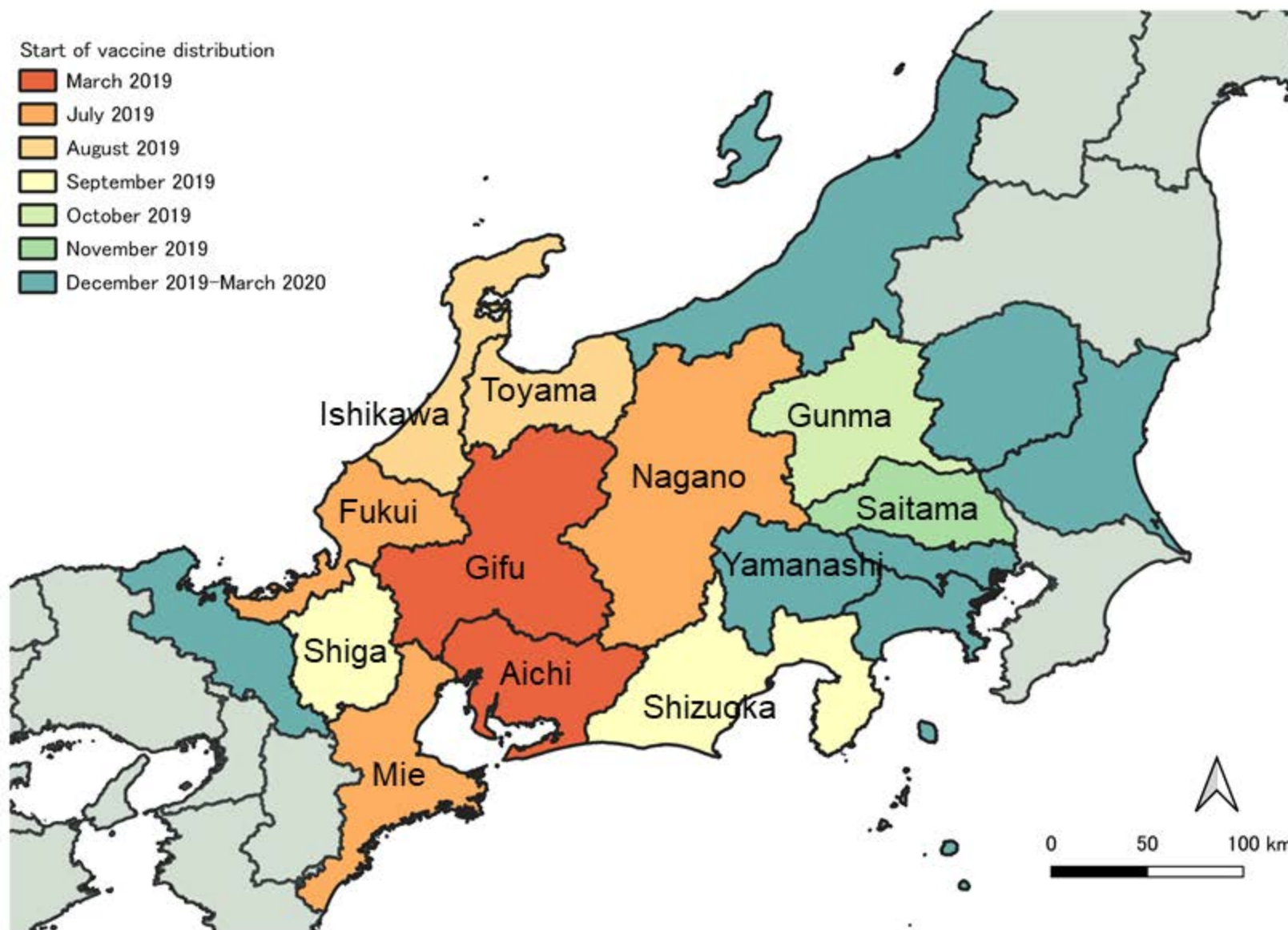

Supplement: Supplementary file 1 — Additional file 1: Supplementary Fig. 1. Distribution of oral bait vaccines. The prefectures where vaccines were distributed were gradually expanded according to the CSF spread in the areas. [file 12917_2021_2891_MOESM1_ESM.pdf]

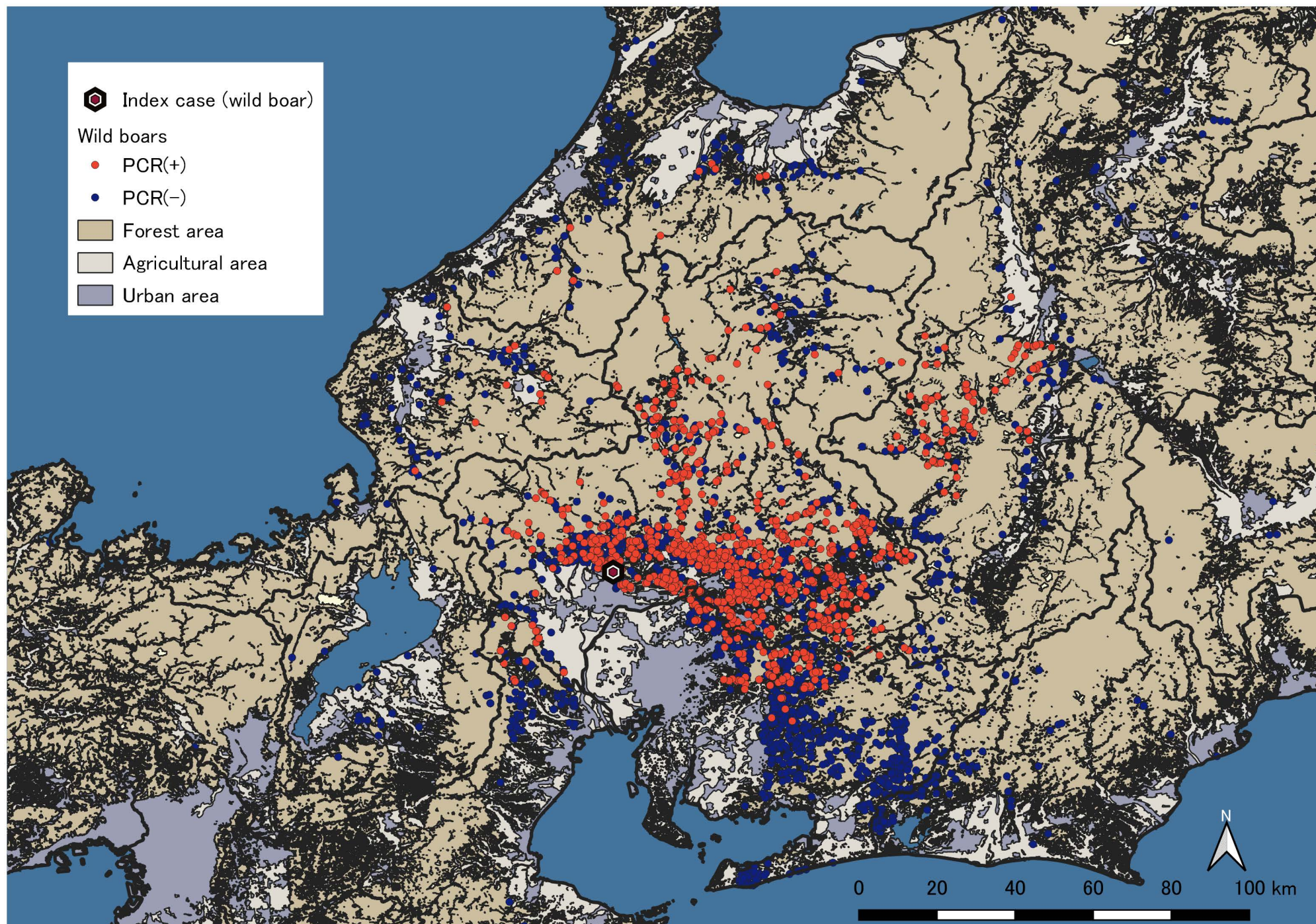

Supplement: Supplementary file 2 — Additional file 2: Supplementary Fig. 2. Geographic information on the area surrounding the index case of a CSF-infected wild boar. Dots indicate the location and PCR test results of wild boars found dead or captured from September 2018 to August 2019 (the period for spread velocity analysis). [file 12917_2021_2891_MOESM2_ESM.pdf]
